# Why are organisational approvals needed for low-risk staff studies in the UK? Procedures, barriers, and burdens

**DOI:** 10.1186/s12913-024-11886-0

**Published:** 2024-11-15

**Authors:** Lesley Dunleavy, Ruth Board, Seamus Coyle, Andrew Dickman, John Ellershaw, Amy Gadoud, Jaime Halvorsen, Nick Hulbert-Williams, Liz Lightbody, Stephen Mason, Amara Callistus Nwosu, Andrea Partridge, Sheila Payne, Nancy Preston, Brooke Swash, Vanessa Taylor, Catherine Walshe

**Affiliations:** 1 International Observatory On End of Life Care, Division of Health Research, Lancaster University, Lancaster, LA1 4AT UK; 2 Department of Oncology, Lancashire Teaching Hospitals NHS Foundation Trust, Preston, PR2 9HT UK; 3 Department of Palliative Medicine, The Clatterbridge Cancer Centre NHS Foundation Trust, Liverpool, L7 8YA UK; 4 Academic Palliative and End of Life Care Centre, Liverpool University Hospitals NHS Foundation Trust, Prescot Street, Liverpool, L7 8XP UK; 5 Palliative Care Unit, Room G036 North West Cancer Research Centre, University of Liverpool, 200 London Road, Liverpool, L3 9TA UK; 6 Lancaster Medical School, Lancaster University, Lancaster, LA1 4AT UK; 7 NIHR Clinical Research Network North West Coast, Liverpool, L3 5TF UK; 8 Department of Psychology, Edge Hill University, Ormskirk, L39 4QP UK; 9 School of Nursing, University of Central Lancashire, Preston, PR1 2HE UK; 10 School of Psychology, University of Chester, Chester, CH1 4BJ UK; 11 School of Human and Health Sciences, University of Huddersfield, Huddersfield, HD1 3DH UK

**Keywords:** Surveys and questionnaires, Research, Health services research, Methodological studies

## Abstract

**Background:**

Health care staff should be given the opportunity to participate in research, but recruiting clinicians via their employing organisation is not always straightforward or quick in the UK. Unlike many countries outside the UK, very low-risk survey, interview or focus group studies can be subject to some of the same governance approval procedures as interventional studies. An exemplar study carried out by the NIHR funded Palliative Care Research Partnership North West Coast is used to highlight the challenges still faced by researchers and health care organisations when setting up a low-risk staff study across multiple NHS and non-NHS sites.

**Methods:**

A study database was created and information was collected on the first point of contact with the clinical site, Health Research Authority (HRA) and local organisational approval times, time from trust or hospice agreement to the first survey participant recruited and overall site survey recruitment numbers. Descriptive statistics (median, range) were used to analyse these data.

**Results:**

Across participating NHS trusts, it took a median of 147.5 days (range 99–195) from initial contact with the local collaborator to recruitment of the first survey participant and hospice sites mirrored these lengthy timescales (median 142 days, range 110–202). The lengthiest delays in the HRA approval process were the period between asking NHS trusts to assess whether they had capacity and capability to support the research and them granting local agreement. Local approval times varied between trusts and settings which may indicate organisations are applying national complex guidance differently.

**Conclusions:**

There is the potential for HRA processes to use more NHS resources than the research study itself when recruiting to a low-risk staff study across multiple organisations. There is a need to reduce unnecessary administrative burden and bureaucracy to give clinicians and research staff more opportunities to participate in research, and to free up NHS R&D departments, research nurses and clinicians to focus on more demanding and patient focused research studies. Hospices need standardised guidance on how to assess the risk of being involved in low-risk research without adopting the unnecessarily complex systems that are currently used within the NHS.

**Supplementary Information:**

The online version contains supplementary material available at 10.1186/s12913-024-11886-0.

## Introduction

Surveys, interviews and focus groups involving health care staff are common ways of collecting important information about practice experiences, care processes, and other key healthcare issues. Accordingly, healthcare staff should be given opportunities to participate in research that has the potential to improve their professional development, working conditions and the care they provide to patients and carers. However, recruiting health care staff in the UK to take part in research via their employing organisation is not always easy, straightforward, or quick [1]. This can be the case even for very low risk staff surveys, interviews or focus group studies [2], which are often subject to some of the same governance approval procedures as higher risk interventional studies. This is in contrast to many countries outside the UK where governance procedures for research involving health care staff, and non-clinical trials of investigational medical products (non-CTIMPs), are less stringent [3].

To avoid or limit such bureaucracy, some survey, interview and focus group invitations are distributed via social media or professional associations and networks [4–6]. This potentially risks excluding some respondents and may skew or bias participation in unknown ways. It is important that recruiting for studies via employing organisations is normalised, with proportionate approvals to reduce the barriers associated with this form of recruitment.

All project based research taking place in the NHS in England and Wales is required to obtain Health Research Authority (HRA) and Health and Care Research Wales (HCRW) approval [7]. There have been recent attempts by the Health Research Authority to try and reduce the administrative burden of the study approval process to improve efficiency and facilitate cross organisational working [2, 8, 9]. The approval process and timelines for a low-risk exemplar staff survey and working group study that required minimal organisational input and resources is described in this paper. The purpose is to highlight the current challenges and barriers still faced by researchers and health care organisations when trying to set up a low-risk staff study across multiple NHS and non-NHS research sites. The exemplar study was set up and carried out in 2021–2022 by the National Institute for Health Research funded Palliative Care Research Partnership North West Coast (PalCaRe-NWC) [10, 11] and a summary of the study design is outlined in Table 1.

**Table 1:** Summary of the exemplar study design

***Research question***: What are the barriers and facilitators to conducting palliative and end-of-life care research in areas of North West England?
***Design***: Descriptive, observational study, including a cross-sectional online survey and working groups using a nominal group technique [12].
***Setting***: The UK NIHR North West Coast region of England (incorporating South Cumbria, Lancashire, Cheshire, and Merseyside).
***Study Population***: All those who had any interest in the provision of, or research into, generalist or specialist palliative care across the region including acute and community NHS Trusts, GP practices, voluntary hospices, other community and private providers of care, clinical research networks, and academic settings. The inclusion criteria asked whether the potential respondent provided health and/or social care for patients and carers (adults and/or children) with palliative care/end of life care needs within the geographical area of interest, and/or is involved or would wish to be involved in palliative/end of life care research.
***Survey Recruitment***: Via local collaborators in NHS Trusts, Hospices, and the North West Coast Clinical Research network. It was also widely disseminated through a project website, personal networks and social media (Twitter, Facebook and LinkedIn).
***Working Group Recruitment***: Survey participants who expressed an interest in taking part were sent invitation packs. Social media (Twitter, Facebook, and Instagram) was also used to advertise the working groups and local collaborators listed above circulated invitation packs.
***Survey data collection***: Local collaborators were asked to send out a survey link via email to eligible staff. In the survey, both closed and free-text questions were used, together with skip options dependent on given answers. The survey identified current and desired levels of palliative care research involvement, current research barriers, suggestions for sustainable solutions and research training needs (See supplementary materials (S1)). The survey was open from 02/03/2022 to 08/06/2022.
***Working group data collection***: Four, two-hour online (via Microsoft Teams) working groups took place. The stages of nominal group technique were followed that included; introductions, silent generation of ideas, listing of ideas, discussion of ideas, ranking of top ten ideas, voting on top ten ideas, discussion of voting and conclusions [12].
***Survey response***: The survey received 495 visitors, of whom 22 declared they did not meet the inclusion criteria, 36 provided no data, and 158 did not proceed beyond the screening questions. Valid responses were received from 293 participants (59.2% of visitors), with 171 of the 293 (58.4%) recording 100% survey progress, and a mean progress of 82.4% (range 100% to 25%).
***Working group response***: Twenty palliative care providers/research staff participated in the working groups.
***Ethics***: Approval was granted by the East of England—Cambridge South Research Ethics Committee (Ref: 22/EE/0049) on the 24/02/2022. NHS research ethics approval was required as we planned to recruit patient and carer participants to a working group.

## Methods

As part of our approach to efficient study management, we maintained a database to track the progress of organisations through the contact and approvals processes pertinent to their organisation. We collected information on our first point of contact with the clinical site, Health Research Authority and local organisational approval times, time from trust or hospice agreement to the first survey participant recruited and overall site survey recruitment numbers. We were interested in exactly how long each approval stage had taken, and the overall timescale from the initial approach to the site, to the study distribution and recruitment of the first survey participant. Given the range of organisations invited to distribute study invitations to their employees, we wanted to know if there were any trends in approval times for different forms of organisation. Descriptive statistics (median, range) were used to analyse these data.

## Results

The National Institute for Health Research North West Coast region of England covers a large geographical area that includes South Cumbria, Lancashire, Cheshire, and Merseyside, with 20 NHS Trusts and 17 hospices (see Table 3 for site recruitment numbers).

### Health Research Authority processes for obtaining local NHS trust approval

**Fig. 1:**
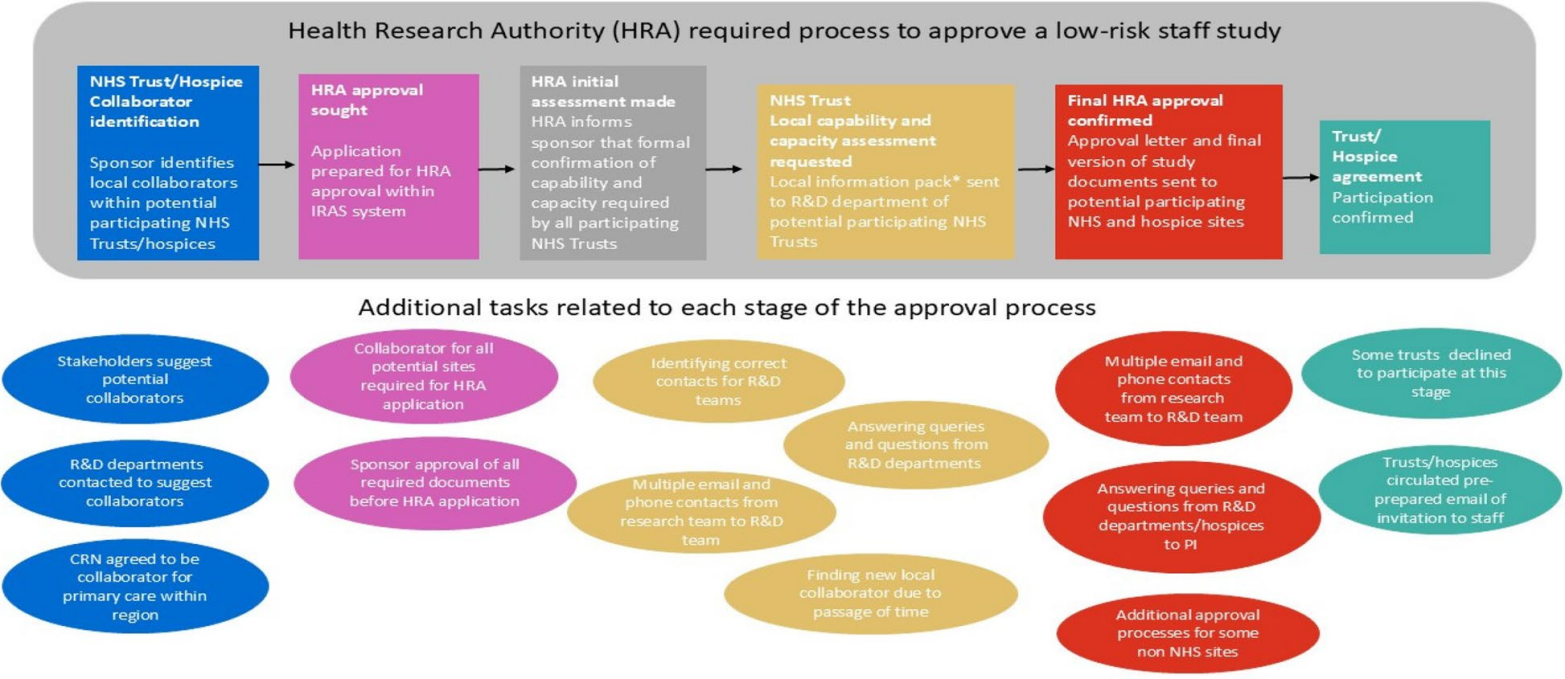
Processes required to gain NHS Trust or hospice approval for a low risk staff study. *A ‘local information pack’ for a non-commercial study contains; study documents such as protocol, participant information, IRAS form and HRA letters, and documents that outline what activities and resources are required by the organisations taking part

Health Research Authority processes that need to be followed to obtain local NHS organisational approval for low risk staff studies are outlined in Fig. 1. For the majority of trusts asked to participate in this research, the only request was to circulate a link to staff to complete an online survey and, in some instances, also to distribute an invitation to staff to take part in a working group. There was no patient recruitment required or research nurse resource needed. The only clinical involvement needed was if the local collaborator circulated the emails themselves and if a staff member agreed to take part in a working group online. We stressed to the organisations taking part that we would only be recruiting small numbers of staff to the working groups from NHS Trusts and hospices across the North West Coast area so the burden for individual organisations would be minimal. We separately identified one trust where we hoped to recruit and consent a small number of patient and carer participants for a single working group discussion, in an attempt to prevent approval delays.

Despite the low-risk nature of the study and national procedures being followed, across NHS trusts it took a median of 147.5 days (range 99–195 days) from the initial contact with the local collaborator to recruitment of the first survey participant. The timelines outlined in Table 2 highlight clearly where the delays occurred within the local NHS organisational approval process required by the Health Research Authority. A collaborator was largely identified within a few days across acute and community trusts demonstrating their willingness to support the study despite clinical pressures. Central Health Research Authority Approval was granted within 25 days, and this also included a review of the study by an NHS research ethics committee.

**Table 2:**
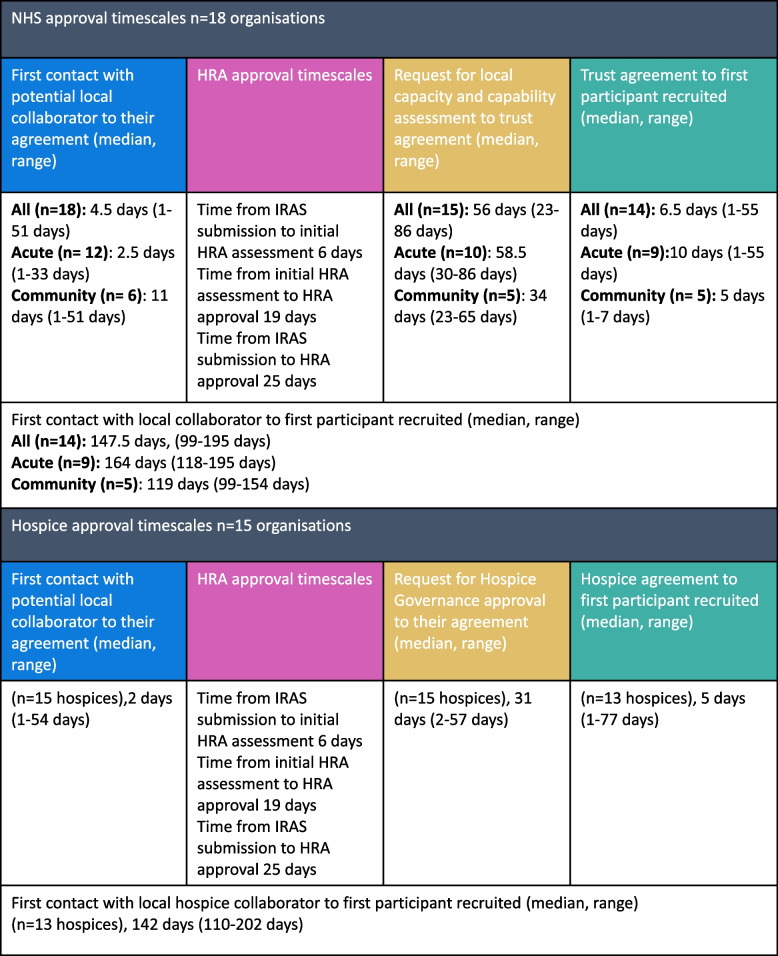
NHS and Hospice approval times

The period between asking NHS trusts to assess whether they had capacity and capability to support the research and them granting local agreement took a median of 56 days (range 23–86 days). The reasons for the delays and the strategies used by the research team to manage and expedite this stage of the process are outlined in Fig. 1. Different processes were sometimes applied within the NHS after central Health Research Authority approval had been granted. For example, the North West Coast Clinical Research Network was able to circulate the survey email to primary care sites on the same day that Health Research Authority Approval was received. Three NHS Trusts granted approval within a few days as the R&D manager or medical director pragmatically reviewed the study documentation. The time from trust agreement to first survey participant recruited took a median of 6.5 days (range 1–55 days). Variations in first survey participant recruitment timescales may have been due to delays in the local collaborator circulating the recruitment email.

### Non-NHS hospice site approval

Heath Research Authority organisational approval does not apply to non-NHS sites, such as independent hospices, and researchers are required to liaise with these organisations individually to obtain their agreement (see Fig. 1). Hospice processes differed and approval could be granted in a variety of ways including by the CEO, clinical lead, clinical governance/quality group, Trustee sub-committee, and in one instance, by the local NHS trust where the hospice was based. Across participating hospices, it took a median of 142 days (range 110–202 days) from the initial contact with the local collaborator to recruitment of the first survey participant, mirroring the lengthy timescales in the NHS. It is important to note that hospice sites needed to be listed on the NHS Integrated Research Application System (IRAS) ethics application, and they had to wait for NHS research ethics approval (IRAS) and the parallel Health Research Authority approval before approving the study (see Fig. 1). As in the NHS sites, a collaborator was largely identified within days and the stage of the process where delays occurred was from the request for approval to their final agreement. How long this process took varied across hospices, due to inconsistencies in how each hospice assessed risk and approved the study as well as other factors such as staff sickness and the impact of COVID 19 pressures on small healthcare organisations. The time from hospice agreement to first survey participant recruited took a median of 5 days (range 1–77 days) so was similar to NHS trusts overall.

### Organisational and participant recruitment

Some organisations were unable to take part because of stated clinical workload and capacity issues, including in NHS R&D departments (see Table 3). This was unexpected as the study was low risk and minimal organisational input and resources were required. Community trusts recruited a median of 17 participants (range 3–19); acute trusts recruited a median of 3 participants (range 0–33) and hospices recruited a median of 5 participants (range 0–20), as shown in Table 3. In one acute NHS trust and two hospices no participants were recruited to the survey despite formally agreeing to support the study.

**Table 3:** Organisation and survey participant recruitment numbers

Setting	Number of organisations approached initially	Number of organisations sent local information pack and/or HRA approval letter	Number of organisations that sent the survey recruitment email	Number of survey participants recruited per type of organisation^a^ (median, range)
Acute	*n*=15 organisations *n*=1 declined because of clinical workload *n*=1 R&D department was unable to identify anyone due to capacity issues. *n*=1 no response	*n*=12 organisations *n*=1 no response after being named on IRAS submission *n*=1 declined due to research nurse capacity issues	*n*=10 organisations	*n*=3 participants (0-33 participants)
Community	*n*=7 organisations *n*=1 declined because of clinical workload	*n*=6 organisations *n*=1 no response after being named on IRAS submission	*n*=5 organisations	*n*=17 participants (3-19 participants)
Hospices	*n*=17 organisations *n*=1 declined because they were providing a reduced service *n*=1 no response	*n*=15 organisations	*n*=15 organisations	*n*=5 participants (0-20 participants)

^a^This figure includes participants that may have been recruited via the project website, personal networks and social media (Twitter, Facebook and LinkedIn) as respondents were only asked to indicate their employing organisation in the survey and not recruitment method

## Discussion

Findings from this exemplar study show that current Health Research Authority approval processes for low-risk staff studies are complex and time consuming for both researchers and health care organisations. It took a median of 147.5 days from initial contact with the local collaborator to the first survey participant being recruited across the NHS Trusts involved in this study. The step in the Heath Research Authority process that had the lengthiest delays was the period between asking NHS trusts to assess whether they had capacity and capability to support the research and them granting local agreement. Local approval times did vary between NHS trusts and settings which may indicate organisations are interpreting and applying complex national guidance differently. Hospices are also influenced by Health Research Authority processes as NHS research ethics committee approval needed to be in place before they could approve the study. There were still hospice approval delays even when confirmation of NHS research ethics committee approval had been received.

It is important that researchers quantify their often hidden experiences of the organisational approval process so that concerns can be raised and areas for improvement be suggested [1]. Health Research Authority approval processes for staff surveys and working groups recruiting from multiple organisations are similar to those used for research studies and clinical trials involving patients. The administrative burden of obtaining organisational approval for low-risk staff research increases the workload of R&D staff, researchers and clinicians, and can contribute to study delays and potentially increase costs [13]. The implementation of current Health Research Authority approval processes for low risk staff research has the potential to use more NHS organisational resources than the research study itself. It is concerning that public and charitable funds are currently being used to cover the time researchers spend obtaining approvals and any resultant study delays.

One way to address this imbalance would be to remove the need for NHS organisations to locally approve low risk staff studies if they have already been reviewed nationally by the Health Research Authority and a research ethics committee. A local collaborator would still need to be identified to support the study and circulate study information. This would allow staff to decide individually if they have ‘capacity and capability’ to take part in a low risk research study. This may require a discussion with their line manager and/or wider team if time away from practice is required. R&D departments could register for audit purposes that their trust is taking part in the research. The need to minimise local governance approval processes in the UK to increase efficiency and reduce waste has already been recognised [1, 13, 14].

There have been growing concerns about research capacity in the NHS, as services are stretched, and care organisations are adjusting to the impact of the COVID pandemic. These issues may have influenced study approval times and recruitment numbers in our study as clinical staff may have had limited capacity to participate in research. The National Institute for Health Research launched the ‘Research Recovery and Reset’ initiative because of the number of studies struggling to meet recruitment targets within acceptable and agreed timescales [15]. It achieved its goal of 80% of NIHR portfolio studies delivering to time and target by June 2023 with 82% of studies meeting this performance indicator in August 2024 [16], but it is now focusing on delays to study set up. In August 2023, 57 commercial and 114 non-commercial portfolio studies were delayed opening by more than 90 days [17]. More recently in August 2024, only 19% of studies sponsored and fully funded by the life sciences industry were open to recruitment within 60 days of HRA approval letter being issued [16]. An independent government review has also taken place to explore why fewer commercial studies are taking place in the UK, leaving patients unable to access innovative treatments [18]. It highlighted how UK approval processes are slow and bureaucratic, especially compared to other countries. There is clearly a need to reduce duplication of effort, unnecessary administrative burden, and improve efficiency when setting up a low-risk staff study. This will give research and clinical staff greater flexibility and more opportunity to take part in important research that has the potential to enhance their development and the care they provide. It will also free up NHS R&D departments, research nurses and clinicians to focus on more demanding and patient focused research studies.

Most hospices are standalone voluntary organisations and, unlike the NHS they often do not have a formal infrastructure in place to support research activity [13]. There is limited guidance for hospices on how to assess the burdens and risk of being involved in research. There is a need for the hospice sector to introduce standardised guidance for approving low risk staff studies, including ensuring research ethics approval is in place, but it is important that they do not adopt the unnecessarily complex systems that are currently used within the NHS.

## Conclusions

Obtaining organisational approval in the UK for low-risk multi-centre staff research is slow and administratively burdensome. There is a need to reduce unnecessary bureaucracy to give clinicians and research staff more opportunities to take part in research. This will also save resources and improve efficiency so that NHS R&D departments and clinicians can be freed up to focus on more demanding and patient focused research studies. Standardised guidance on how hospices should assess the risk of being involved in low-risk research should be developed, without adopting the complex systems that are currently used within the NHS.

## Supplementary Information

Supplementary Material 1.

## Data Availability

Data are stored in Lancaster University’s PURE repository. The authors may be contacted to obtain further clarifications on aspects of the study not provided in this paper or supplementary material wherever ethically or legally appropriate.
