# Supplementary material for: Why are organisational approvals needed for low-risk staff studies in the UK? Procedures, barriers, and burdens
Source: BMC Health Serv Res. 2024 Nov 15;24:1408. doi: 10.1186/s12913-024-11886-0 (PMC11566500; doi:10.1186/s12913-024-11886-0)
Supplement: Supplementary file 1 — Supplementary Material 1. [file 12913_2024_11886_MOESM1_ESM.docx]

#

**Why are organisational approvals needed for low-risk staff studies in the UK? Procedures, barriers, and burdens.**

# Supplementary File 1. Survey

Thank you for your interest in completing this survey. Research is an important part of excellent care, with health and social care organisations involved in research activity known to provide better care. However, in the North West Coast area of England we know that research activity in the important field of palliative and end-of-life care is much lower than in other parts of the country. This is despite much higher needs than on average. It is important that we invest in research in North West Coast England to ensure that the particular needs of the populations in this area are properly taken account of, and that care is planned and provided to meet these needs in the best possible way. We are trying to find out what the main barriers are to palliative care research locally, and how we can best overcome these to meet the needs of those in the North West Coast area. This study is funded by the National Institute for Health Research.

We realise you are very busy right now, and so we have tried to balance collecting the information, with keeping the questionnaire as short as we can. The survey has 5 sections, and should take no longer than 15-20 minutes to complete, although it may depend on how much additional/open comments you wish to share.

- We ask that this survey be completed by all those who may have an interest in palliative care research as a care provider or researcher/research staff within the North West Coast area of England.
- You may provide health and/or social care for patients and carers with palliative care/end of care needs in the North West Coast area of England **and/or** be involved or would wish to be involved in palliative care/end of life care research.*

** (This does not only include practitioners who provide specialist palliative care but also those that provide generalist palliative care in hospitals, primary care and nursing/care homes (e.g. DNs, GPs, Consultants, Physios etc) for adults and/or children.* *It also includes research focused staff (e.g. research nurses, researchers, R&D staff).)*

Participation in the survey is voluntary. We will consider everything that you say. Your reply will help us. We will share the results of this survey through publications and presentations. The results will be aggregated and anonymised so no-one should be able to tell which individual has provided particular information. We ask which organisation you work for to provide recruitment data to the National Institute for Health Research but this will be anonymised when shared. We do not think there are particular risks to completing this survey. Completion of this survey implies consent for your data to be used as part of this study. You will input your data into a secure online survey platform, and these data will be then stored in a secure institutional filestore at Lancaster University.

If you wish to speak to anyone about this survey you can contact Lesley Dunleavy Senior Research Associate ([l.dunleavy@lancaster.ac.uk](mailto:l.dunleavy@lancaster.ac.uk)). You may also contact her if you wish to withdraw your responses, up to 2 weeks following completion. If you have made a partial response we may contact you after a week to check if this is an error.

This study has been granted NHS research ethics approval by East of England - Cambridge South Research Ethics Committee (Ref: 22/EE/0049). If you wish to speak with someone independently about this research you can contact the Director of Research Professor Fiona Lobban ([f.lobban@lancaster.ac.uk](mailto:f.lobban@lancaster.ac.uk)).

If you are happy to proceed, please consent to participate by clicking below which will take you to the first page of the survey.

SCREENING QUESTION:

| Do you provide health and/or social care for patients and carers (adults and/or children) with palliative care/end of care needs in the North West Coast area of England? (This area covers Cumbria, Lancashire, Cheshire and Merseyside)  **and/or** is involved or would wish to be involved in palliative/end of life care research?*  * This does not only include practitioners who provide specialist palliative care but also those that provide generalist palliative care in hospitals, primary care and nursing/care homes. It also includes research focused staff (e.g. research nurses, researchers, R&D staff). | Yes  No  IF NO then survey finishes |
| --- | --- |

INFORMATION ABOUT THE PERSON COMPLETING THE SURVEY

This information will only be used in case we need to check back with you, for example if the survey does not save correctly. This information will be stored separately to the data that you provide, to adhere to GDPR and maintain confidentiality and anonymity.

| What is your name? | Free text |
| --- | --- |
| What is your contact email address? | Free text |

**A. Personal Information**

This section of the survey aims to gather background information about those who are participating in this research.

1. **How long have you worked in palliative care?** This may include specialist palliative care, generalist palliative care or palliative care research roles. Please select one.

- Just getting started (<2 years)
- Early career (2-5 years)
- Mid-career (6-10 years)
- Late career (10+ years)

1. **Please provide the name of the organisation that you work for (name of trust/hospice/university etc)?**

**______________________________________________________________________**

**3 What is your primary professional role?** If more than one applies, please choose your primary role.

- Nurse
- Doctor
- Social Worker
- Psychologist
- Occupational Therapist
- Physiotherapist
- Manager/admin
- Other (please specify): ____________________________

1. **What is your primary work environment?** If more than one applies, please choose your primary work environment.

- University
- Hospital
- Primary Care
- Hospice
- Nursing home and/or care home
- Clinical Research Network
- NHS R&D department
- Other (please specify): ____________________________

**5 If your role is primarily clinical (rather than research) do you work in specialist palliative care or do you provide general palliative care as part of a wider caseload?**

- Specialist palliative care
- Generalist palliative care
- Work only in research role
- Other primary role. Please specify____________________

**6 Is your work (clinical or research) primarily with adults or children?**

- I work primarily with adults
- I work primarily with children

**B. Current and desired level of palliative care research involvement**

This section seeks to understand your current level of involvement in palliative care research as well as your desire for continued or further research involvement.

1. **What is your weekly full time equivalent (FTE)? This is across all your roles if you have a joint or shared appointment? (e.g. 1 day a week = 0.2 FTE, 2 days a week = 0.4 FTE, 3 days a week= 0.6 FTE, 4 days a week = 0.8 FTE, full time = 1.0 FTE)______________**
2. **What proportion of your average working week is spent on palliative care research?**

Please select one.

- Currently I do not spend any time on palliative care research
- Less than 10%
- 10-25 %
- 26-50%
- 51-75%
- 76-100%

1. **Would you like to increase your involvement in palliative care research?** Please select one.

- Yes
- No

1. **Please indicate which of the following four categories most accurately describes your palliative care research experience?** Please select the most appropriate category.

- Non-active: Little or no previous experience and currently not participating in palliative care research activities.
- Involved: Involved as part of a team delivering palliative care research.
- Managing: Managing own palliative care projects as a ‘clinician researcher’ or as an academic.
- Supervising: Supervising the research activities of others.

1. **How many funded palliative care research projects are you currently involved in?** Please select one.

- None
- 1-3
- 4-7
- 8-10
- More than 10

**C. Barriers to participating in palliative care research within North West Coast**

This section relates to developing an understanding of the barriers to participation in palliative care research encountered by practitioners and research focused staff within North West Coast.

1. **To what extent are the following factors a barrier to your participation in palliative care research within North West Coast?** Please rate each of the following items based on your evaluation of the degree to which it hinders your ability to participate in palliative care research at the moment.

|  | **Not a barrier** | **Minor barrier** | **Moderate barrier** | **Significant barrier** |
| --- | --- | --- | --- | --- |
| Alignment of palliative care research-related activities with organisation's mission and goals |  |  |  |  |
| Confidence in my abilities to do palliative care research |  |  |  |  |
| No formal palliative care research training |  |  |  |  |
| My understanding of what is needed to conduct palliative care research |  |  |  |  |
| My knowledge and skills to conduct palliative care research |  |  |  |  |
| My work environment (e.g. lack of support from management) |  |  |  |  |
| Palliative care research not perceived as important by my organisation |  |  |  |  |
| Lack of funding or financial resources prevents me from becoming involved in palliative care research |  |  |  |  |
| Understanding how to apply for research funding to support palliative care research |  |  |  |  |
| Lack of protected time/competing demands |  |  |  |  |
| Lack of access to computer facilities |  |  |  |  |
| Lack of access to a statistician |  |  |  |  |
| The time and effort required to initiate a palliative care research project discourages me from the start |  |  |  |  |
| Lack of access to relevant data |  |  |  |  |
| Working with new technologies (i.e. software) intimidates me |  |  |  |  |
| I’m not interested in palliative care research |  |  |  |  |
| I do not know how to get involved in palliative care research |  |  |  |  |
| Lack research ethics committee/ethics expertise |  |  |  |  |
| No access to palliative care research collaborators/partners |  |  |  |  |
| Lack of partnership agreements for sponsorship and indemnity |  |  |  |  |
| Loss of clinical productivity during palliative care research activities |  |  |  |  |
| Loss of income during palliative care research activities |  |  |  |  |
| Palliative care research is not worth the time and resource investment |  |  |  |  |
| Difficulty balancing working independently and knowing when to ask someone for assistance |  |  |  |  |
| Inability to find the right people to ask the right questions |  |  |  |  |
| No opportunities to interact with palliative care researchers |  |  |  |  |
|  |  |  |  |  |

1. **Please use the space below to help us understand more about the barriers and issues that affect your involvement in palliative care research. You can add additional barriers or issues not listed above, or give more details about the barriers you think are particularly important.**

**________________________________________________________________________________________________________________________________________________________________________________________________________________________**

**D. Perceptions of activities that would facilitate participation in palliative care research within North West Coast**

This section seeks to understand the activities that you believe would facilitate your participation in palliative care research within North West Coast.

1. **What would facilitate your participation in palliative care research within North West Coast?** Please indicate below the extent to which you agree that each activity would enhance your participation in palliative care research within North West Coast.

|  | **Strongly agree** | **Agree** | **Disagree** | **Strongly disagree** |
| --- | --- | --- | --- | --- |
| Palliative care research mentors’ programme |  |  |  |  |
| Palliative care research information network |  |  |  |  |
| More exposure to palliative care research e.g. via an internship |  |  |  |  |
| More exposure to palliative care research as undergraduates |  |  |  |  |
| Palliative care research seminars for those in practice |  |  |  |  |
| Attending research conferences |  |  |  |  |
| Supportive management |  |  |  |  |
| Staff cover |  |  |  |  |
| Access to funding to support research |  |  |  |  |
| Palliative care research is included as part of my job description |  |  |  |  |
| Collaboration with other centres |  |  |  |  |
| Availability of resources such as a guide/manual |  |  |  |  |
| Ongoing research working group at work |  |  |  |  |
| Availability of workshops for writing proposals |  |  |  |  |
| Availability of workshops for statistics |  |  |  |  |
| Availability of workshops for study design |  |  |  |  |
| Availability of workshops for developing research questions |  |  |  |  |
| Availability of workshops for writing for publication |  |  |  |  |
| Assistance with getting started with research |  |  |  |  |
| Statistics advisor |  |  |  |  |
| Availability of research policies and governance procedures to support palliative care research |  |  |  |  |
| Access to allocated research staff |  |  |  |  |
| Supportive clinical lead |  |  |  |  |
| Access to Patient and Public Involvement (PPI) resources or input |  |  |  |  |
| Access to palliative care research recruitment training |  |  |  |  |
|  |  |  |  |  |

**2 Please use the space below to help us understand more about the facilitators that would affect your involvement in palliative care research. You can add additional facilitators not listed above, or give more details about the facilitators you think are particularly important.**

**____________________________________________________________________**

**____________________________________________________________________**

**_____________________________________________________________________**

**_____________________________________________________________________**

**E. Research training needs**

In order to perform palliative care research effectively you need relevant skills. This section seeks to explore knowledge gaps that you may have with regards to conducting palliative care research in order to identify your research training needs. This includes the types of training, assistance, or resources that you believe is required in order for you to safely and competently engage in palliative care research initiatives. Please answer all the questions as honestly as possible to enable us to compile a complete picture of your training requirements.

1. **Have you received any palliative care research-related training in the past 3 years?**

- Yes
- No

1. **What kinds of training, technical assistance, or resources would you be interested in receiving to support your participation in palliative care research?** Please rate each of the following research activities you would be interested in receiving support for from least interested to most interested.

|  | **Least interested** | **Moderately interested** | **Most Interested** |
| --- | --- | --- | --- |
| **Research processes** |  |  |  |
| 1. Conducting literature reviews |  |  |  |
| 1. How to design rigorous and evidence-based research while being pragmatic and taking into account the complex environment in which palliative care research is often carried out. |  |  |  |
| 1. Research question and hypothesis generation (if appropriate) |  |  |  |
| 1. Selecting appropriate conceptual/theoretical frameworks |  |  |  |
| 1. Designing palliative care research studies |  |  |  |
| 1. Identifying relevant variables |  |  |  |
| 1. Selecting the best measures/scales to use |  |  |  |
| 1. Qualitative research designs |  |  |  |
| 1. Obtaining buy-in from staff |  |  |  |
| 1. Determining sample size |  |  |  |
| 1. Recruitment of participants |  |  |  |
| 1. Quantitative data collection |  |  |  |
| 1. Qualitative data collection (e.g., focus groups, interviews) |  |  |  |
| 1. Qualitative data analysis (e.g., analyse interview data) |  |  |  |
| 1. Quantitative data analysis |  |  |  |
| 1. Data entry and data cleaning |  |  |  |
| 1. Using research data to inform programmes and services |  |  |  |
| 1. Project management |  |  |  |
| 1. Developing a programme of research |  |  |  |
| 1. Finding research partners and expert consultation (e.g., biostatistical expertise) |  |  |  |
| 1. Identifying research mentors |  |  |  |
| **Learning specific research related skills** |  |  |  |
| 1. Writing a proposal |  |  |  |
| 1. Finding grant funding |  |  |  |
| 1. Writing a successful grant application |  |  |  |
| 1. Preparing an ethics application |  |  |  |
| 1. Conducting statistical data analyses |  |  |  |
| 1. Writing for publication |  |  |  |
| 1. Developing a budget for a research project |  |  |  |
| 1. Presenting your research at professional meetings |  |  |  |
| 1. Using knowledge dissemination strategies other than publication and conference presentations |  |  |  |
| 1. Technology needs (e.g., information technology/electronic records, audio/video conferencing, computer hardware, statistical software) |  |  |  |
|  |  |  |  |

1. **If we were to offer palliative care research education or training, how would you best like to receive this?** Please select one.

- Online modules
- Face-to-face workshop
- Webinars
- Written resources
- Research mentor
- Other (please specify): _____________________________

**4 List up to three research-related priority areas in which you would like to receive further training.**

____________________________________________________________________________________________________________________________________________________________

____________________________________________________________________________________________________________________________________________________________

______________________________________________________________________________

______________________________________________________________________________

If you are interested in taking part in additional research to explore the barriers and facilitators to palliative care research within North West Coast please indicate below so we can contact you with further information:

- Yes I would like to receive information about further research
- No I would not like to receive information about further research

**Thank you for completing this survey. Your responses are extremely valuable to help us understand the local barriers to palliative and end of life care research within the North West Coast region as well as helping us to identify how they may be overcome. Your time and input is very much appreciated. Please do not hesitate to contact Lesley Dunleavy at** [**l.dunleavy@lancaster.ac.uk**](mailto:l.dunleavy@lancaster.ac.uk) **with any questions or concerns.**
